# Three-dimensional visualization of electroacupuncture-induced activation of brown adipose tissue via sympathetic innervation in PCOS rats

**DOI:** 10.1186/s13020-022-00603-w

**Published:** 2022-04-18

**Authors:** Hongru Gao, Xiaoyu Tong, Wei Hu, Yicong Wang, Kuinyu Lee, Xiaoqing Xu, Jiemei Shi, Zhenle Pei, Wenhan Lu, Yuning Chen, Ruonan Zhang, Zheyi Wang, Ziyu Wang, Chengzhi Han, Yu Wang, Yi Feng

**Affiliations:** State Key Laboratory of Medical Neurobiology, Department of Integrative Medicine and Neurobiology, School of Basic Medical Sciences, Institutes of Brain Science, Brain Science Collaborative Innovation Center, Fudan Institutes of Integrative Medicine, Fudan University, Shanghai, 200032 China

**Keywords:** Polycystic ovary syndrome, Electroacupuncture, Brown adipose tissue, Sympathetic innervation, Uncoupling protein 1

## Abstract

**Background:**

Low-frequency electroacupuncture (EA) has been shown to ameliorate obesity and reproductive dysfunctions in patients with polycystic ovary syndrome (PCOS), and further explorations in PCOS-like rats showed that EA could affect white adipose tissue. However, the function and neuromodulation of brown adipose tissue (BAT) in PCOS and after EA treatment have remained unknown. The present study focused on the role of BAT in PCOS-like rats and its relationship with EA and characterized the three-dimensional (3D) innervation of BAT associated with activation molecules.

**Methods:**

Female rats (21 days old) were implanted with dihydrotestosterone or fed with a high fat diet to establish PCOS-like and obesity models, respectively, and then EA treatment at “Guilai” (ST 29) and “Sanyinjiao” (SP 6) was carried out for 4 weeks. In the present study, morphological analysis, 3D imaging, molecular biology, and other experimental techniques were used to study the sympathetic nerves and activity of BAT.

**Results:**

PCOS-like rats showed both obvious weight gain and reproductive dysfunction, similar to what was seen in obese rats except for the absence of reproductive dysfunction. The body weight gain was mainly caused by an increase in white adipose tissue, and there was an abnormal decrease in BAT. Because both the lipid metabolism and reproductive disorders could be improved with bilateral EA at “Guilai” (ST 29) and “Sanyinjiao” (SP 6), especially the restoration of BAT, we further investigated the neuromodulation and inflammation in BAT and identified the sympathetic marker tyrosine hydroxylase as one of the key factors of sympathetic nerves. Modified adipo-clearing technology and 3D high-resolution imaging showed that crooked or dispersed sympathetic nerves, but not the twisted vasculature, were reconstructed and associated with the activation of BAT and are likely to be the functional target for EA treatment.

**Conclusion:**

Our study highlights the significant role of BAT and its sympathetic innervations in PCOS and in EA therapy.

**Supplementary Information:**

The online version contains supplementary material available at 10.1186/s13020-022-00603-w.

## Introduction

Polycystic ovary syndrome (PCOS) is a complicated and heterogeneous reproductive-endocrine-metabolic disorder in women and is closely associated with obesity, insulin resistance, type 2 diabetes, liver damage, etc. Recent studies suggest that adipose tissues, including white adipose tissue (WAT) and brown adipose tissue (BAT), play important roles in the occurrence, development, and prognosis of PCOS [1, 2]. About half of all women with PCOS suffer from central obesity and overweight [3], and the incidence rates of infertility, cardiovascular disease, diabetes, and non-alcohol fatty liver in PCOS patients with obesity are significantly higher than in lean PCOS patients. PCOS also has a significant impact on the metabolism of the offspring [4–6].

The function of mammalian WAT is to store and release energy, while the function of BAT is to consume energy and produce heat in response to different stimuli. The thermogenic activity of BAT primarily relies on the function of mitochondrial brown fat uncoupling protein 1 (UCP1), which is located on the inner membrane of mitochondria. Clinical trials have demonstrated that BAT activity is weakened in PCOS patients, probably induced by central obesity [7]. BAT transplantation can improve whole body energy metabolism in PCOS rodent models and can rescue the reduction of BAT activity [8, 9]. BAT is innervated by the sympathetic nervous system and can be activated by norepinephrine, which originates from sympathetic nerves and binds to adrenergic receptors expressed in BAT [10, 11].

Acupuncture, a form of traditional Chinese therapy, has a long history of treating gynecological and metabolic disorders [12–14]. In clinical practice, electroacupuncture (EA) has proven to be effective in weight management in PCOS patients [15], and it is suggested that EA can directly affect adipose tissue and stimulate the browning of WAT through UCP1 [16, 17]. However, due to the limitations of traditional techniques, the mechanism through which acupuncture improves endocrine and metabolic disorders has remained unclear.

Advanced three-dimensional (3D) tissue clearing techniques might provide a new way for addressing these questions. Tissue transparency includes a large category of techniques, including CLARITY, CUBIC and PEGASOS, all of which are designed to remove light-scattering lipids. We used the CLARITY and CUBIC methods to observe the blood vessels and innervation of the ovary [18, 19]. However, due to the rich lipid droplets in the adipose tissue, large adipose tissue clearing remained challenging. iDISCO, a novel dehydration/rehydration-based method, allowed for the clearing of adipose tissue. With iDISCO, sympathetic innervations of the interscapular BAT were shown to decrease in the obese mice [20]. In the present study, we modified the immunostaining and clearing methods for BAT and optimized the 3D imaging quality with single cell high spatial-resolution lightsheet microscope in order to investigate the role of sympathetic innervation in BAT in DHT-induced PCOS-like rats and the mechanism of action of EA.

## Materials and methods

### Animals and experimental design

Female Wistar rats (21 days old, Shanghai SLAC Laboratory Animal Co., Ltd., Shanghai, China) were randomly divided into the control, obesity, obesity + EA, PCOS, and PCOS + EA groups (n = 7–8 per group). All rats were housed in 12-h light/dark conditions with constant temperature (22 ± 2 °C) and humidity (45–55%) and with free access to food and water. At postnatal day 21, the obesity model was established by feeding the rats a 60 kcal % fat diet (#D12492, Research Diets, Inc. New Brunswick, USA) for 12 weeks. Rats in the PCOS and PCOS + EA groups were implanted with a sustained-release tube containing 15 mg DHT (Sigma-Aldrich, A8380, USA) to be evenly released over 90 days. The obesity + EA and PCOS + EA rats were given EA treatments from week 9 to week 12 after modeling. The experimental procedures were approved by the local ethics committee of Shanghai Medical College, Fudan University (No. 20130227-024).

### EA treatments

EA treatment lasted for 30 min per day from Monday to Friday (9–11 a.m.) for 4 weeks. Rats were anesthetized briefly by isoflurane (2% in a 1:1 mixture of oxygen and air, RWD Life Science Co., Shenzhen, China) and were suspended on a platform and were conscious during the EA treatment.

Bilateral “Sanyinjiao” (SP6) and bilateral “Guilai” (ST29) were the acupoints used in the EA treatment. “Sanyinjiao” (SP6) and “Guilai” (ST29) were chosen as EA points based on their verified effect on both DHT-induced PCOS-like rat models and in clinical trials [21]. During the EA treatments, the location and depth of selected acupoints were based on the *Atlas of Basic and Clinical Acupuncture* published by Jie Yan and Bing Zhu (Hunan Science and Technology Press, 2010). “Sanyinjiao” (SP6) is located at 10 mm above the tip of the rat medial malleolus. The depth of penetration was 5 mm in the flexor digitorum profundus. “Guilai” (ST 29) is located at 33 mm below the umbilicus and 16.5 mm bilaterally away from the anterior midline. The depth of penetration was 2 mm at the outer edge of the rectus abdominis. Needles (Suzhou Medical Appliance Factory, China) were attached to an electrical stimulator (HANS-LH202, Huayang Co., Ltd., China) at 2 Hz and 1–2 mA. Needles were sterile, and the acupuncture manipulation was performed by one researcher. At the end of the experiment, all rats were sacrificed by deep anesthesia to collect the tissues and serum. The control and obesity groups were sacrificed in the diestrus stage of the estrous cycle.

### Estrous cycle

Vaginal smears were carried out to detect the estrous stage from week 8 after DHT implantation until the end of the experiment. Smears were obtained daily at 4–5 p.m. to determine the cyclicity stage by analyzing the predominant cell type under a microscope. The estrous cycle consists of four stages with specific major cell types: proestrus, including round nucleated epithelial cells; estrus, including cornified squamous epithelial cells; metestrus, including cornified squamous epithelial cells and leukocytes; and diestrus, including nucleated epithelial cells and leukocytes. The representative smears were stained with hematoxylin and eosin (H&E).

### Oral glucose tolerance test (OGTT) and insulin tolerance test (ITT)

The OGTT was performed three days prior to sacrifice. All rats were fasted for at least 12 h while water was accessible, and the second drop of blood from the tail tip was analyzed with a glucometer (ACCU-CHECK Performa, Roche, USA). The tail of the animal was properly covered by gauze after the measurement. The blood glucose level was measured before being given the oral dose of D-glucose (3 g/kg body weight, 50% concentration) and then at 30, 60, 90, and 120 min after the dose.

The ITT was performed one day before sacrifice. The operation was the same as for OGTT, and insulin (0.75 U/Kg) was injected after testing the fasting glucose level and then at 30, 60, 90, 120, and 180 min after the injection. It was necessary to prepare an oral glucose solution in case of hypoglycemia.

### Micro computerized tomography (micro-CT) of BAT

Eight weeks after modeling and at the end of the EA treatment, micro-CT (Quantum; PerkinElmer, Hopkinton, MA, USA) was carried out to acquire images of BAT. The rats were anesthetized continuously by isoflurane (2% in a 1:1 mixture of oxygen and air, RWD Life Science Co., Shenzhen, China) with a mask in the CT machine. The X-ray parameters were 114 µA and 70 kVp. The scanning process was managed by a computer, and the image field was 72 mm × 40 mm with 144 voxels. Analyze 12.0 software (Analyze Direct, KS, USA) was used to acquire the CT images and for image segmentation, and the target area module and volume editing tools in the software were used to quantify the BAT volume.

### Endocrine hormone and metabolic index profile

Serum samples were obtained from the abdominal aorta prior to sacrificing the rats and stored at − 80 ℃ pending enzyme linked immunosorbent assay (ELISA) of 17β-estradiol (E2), testosterone (T), dihydrotestosterone (DHT), sex hormone binding globulin (SHBG), alanine transaminase (ALT), aspartate transaminase (AST), triglyceride (TG), total cholesterol (TC), low-density lipoprotein (LDL), high-density lipoprotein (HDL), free fatty acids (FFA), C-reactive protein (CRP), aldosterone (ALD), chemerin, and leptin. Endogenous hormone and cholesterol levels were measured using radioimmunoassay kits and colorimetric kits (Additional file 1: Table S3) following the manufacturer’s protocols. The results were recorded by a microplate reader (SpectraMax Paradigm, Molecular Devices, USA). Duplicates were made for each sample.

### Quantitative real-time PCR analysis

Total RNA of adipose tissue was isolated by Trizol reagent (9109Q, Takara Bio, Inc. Japan) according to the manufacturer’s instructions, and single-stranded cDNA was synthesized from each sample (2 μg) with PrimeScript RT Master Mix (#RR036A, Takara Bio, Inc., Japan). Quantitative real-time PCR (qRT-PCR) was performed with an ABI PRISM 7900 sequence detection system (Applied Biosystems, Foster City, CA). The PCR parameters were set according to the manufacturer’s protocols, and amplifications were performed with a SYBR Premix Ex Taq kit (#RR420A, Takara Bio, Inc., Japan). For each sample, duplicate reactions were performed in 96-well plates, and all primers were checked to ensure the uniformity of the target gene. Primer quality was further demonstrated by the dissociation curve in the qRT-PCR test prior to use. The primer sequences for Uncoupling Protein 1 (*Ucp1*), Interleukin-1β (*IL-1β*), Interleukin-6 (*IL-6*), and *β-actin* are listed in Additional file 1: Table S1. Relative gene expression was determined with the 2^–∆∆CT^ formula.

### Western blotting analysis

The protein content of the fat tissues was extracted with a kit (BB31226, Bestbio, China), and a BCA kit (23327, Thermo Scientific, Rockford, USA) was used to determine the protein concentration. Equal amounts (20 μg) of protein for each treatment group were resolved using an SDS-PAGE Gel Quick Preparation kit (Beyotime Biotechnology Co, China) and transferred onto PVDF membranes. The membranes were probed with primary antibodies in 0.01 M Tris-buffered saline supplemented with Tween-20 containing 5% bovine serum albumin followed by HRP-conjugated secondary antibody. When necessary, the PVDF membranes were stripped by Western blot stripping buffer (Thermo Scientific, Rockford, USA) for 30 min at room temperature, washed twice in TBST, and then re-probed. The protein bands were detected with an ImageQuant LAS4000 mini-gel imaging system (GE Healthcare Life Sciences, Pittsburgh, USA). All specific protein band densities were normalized to β-tubulin as the loading control and analyzed with Image-Pro Plus 6.0 (Media Cybernetics, USA).

### Adipo-clear and three-dimensional (3D) imaging analysis

Following the modified iDISCO protocol [22], the rats were deeply anesthetized with 20% urethane and fixed with an intracardiac perfusion of ice-cold 0.9% saline and 4% paraformaldehyde. All samples were postfixed overnight at 4 ℃ and then washed with 1 × PBS for 1 h at room temperature three times. The samples were then dehydrated at room temperature in 20%, 40%, 60%, 80% methanol for 1 h and then incubated in 100% methanol for 3 h. Samples were then bleached with 5% H_2_O_2_ in 20% DMSO/methanol (1 vol 30% H_2_O_2_/1 vol DMSO/4 vol methanol, ice cold) at 4 ℃ overnight. After bleaching, the samples were rehydrated in 100%, 80%, 60%, 40%, and 20% methanol and then in PBS for 1 h each and then in PTx.2 (0.2% Triton X-100 in PBS) twice.

Pretreated samples were incubated in permeabilization solution and blocking solution at 37 ℃ for 2 days at most. The samples were then immunolabeled with primary antibody in PBS, 0.2% Triton X-100, 20% DMSO, and 0.3 M glycine at 37 ºC for the indicated time and then washed in PBS and 0.2% Tween-20 with 10 mg/ml heparin for 1 day. Samples were incubated in secondary antibody solutions with PBS, 0.2% Triton X-100, 10% DMSO, and 6% donkey serum at 37 ºC for the indicated time (Table 1) and washed in PBS and 0.2% Tween-20 with 10 mg/ml heparin for 1 day and then dehydrated in 20%, 40%, 60%, 80%, and 100% methanol for 1 h each. Finally, samples were incubated in 66% dichloromethane (Sigma 270997-12X100ML) and 33% methanol for 3 h and in 100% dichloromethane twice for 15 min to wash out the methanol. The samples were immersed in dibenzyl ether (Sigma 108014-1 KG) until clear and then stored in dibenzyl ether at room temperature.

**Table 1:** Incubation times for inmmunostaining

Buffer	Small tissue	Large tissue	Temperature
Primary antibody incubation	3 days	4-5 days	37 ℃
Secondary antibody incubation	3 days	4-5 days	37 ℃

The approximate weight of small tissue is < 300 mg

Cleared adipose tissues were imaged by a light-sheet microscope (LS-18, Nuohai Life Science, Co., Ltd., Shanghai, China) with a 1 × objective and 6.3 ×zoom. The 3D images were analyzed and reconstructed by Imaris (v. 9.0, Bitplane, Oxford, UK). The Imaris Spot algorithm was used to semi-manually determine the identity of UCP1, while the Surface algorithm and Filament algorithm were used to reconstruct the sympathetic innervation.

### Statistical analysis

Data are presented as the mean ± standard error of the mean. The results for three groups were analyzed by one-way ANOVA and two-way ANOVA with Tukey’s test (Prism 8; GraphPad Software, San Diego, CA, USA), and p-values < 0.05 were considered statistically significant.

## Results

### Low-frequency EA reduced body weight while restoring BAT in both PCOS-like and obese rats

**Fig. 1:**
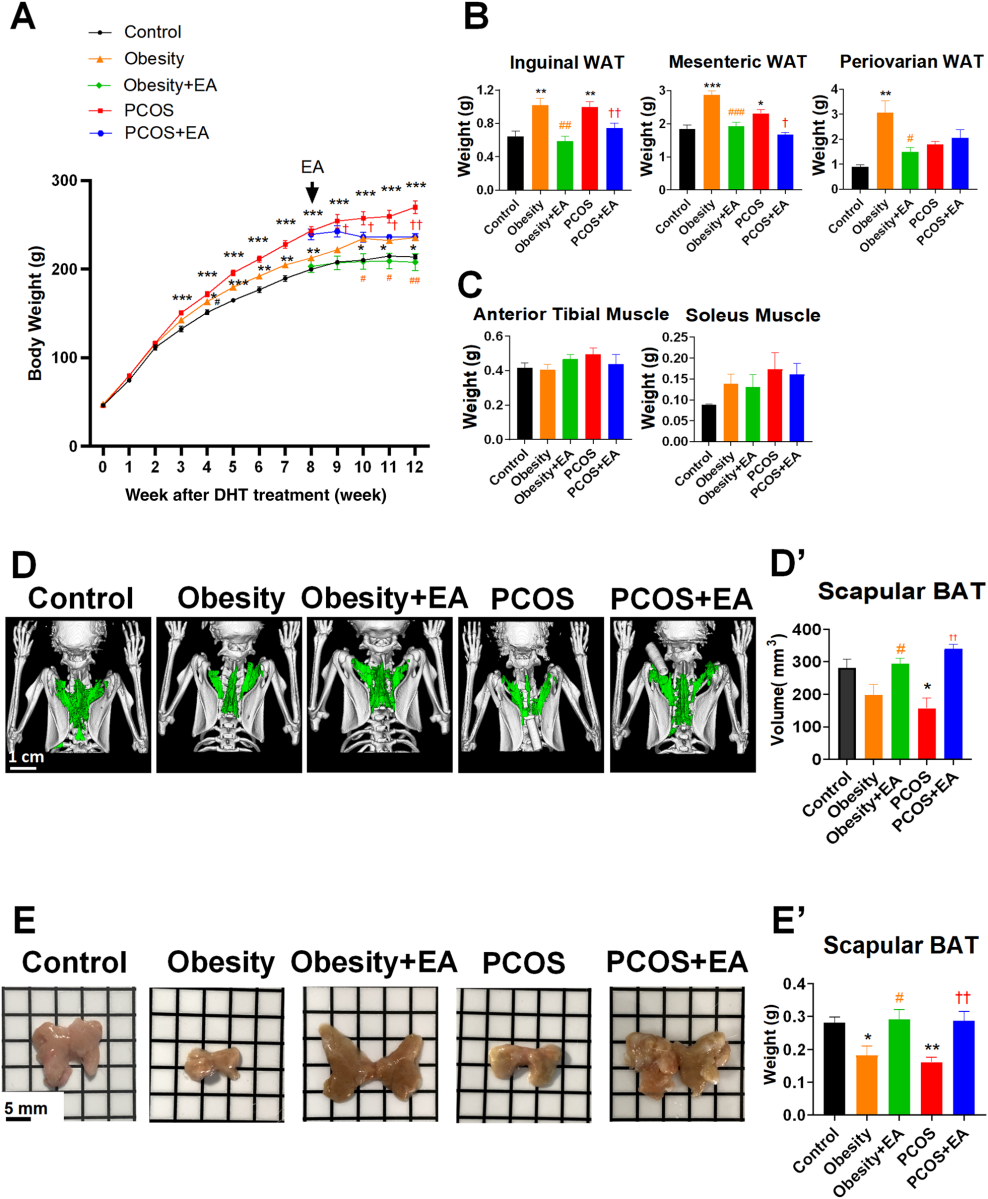
PCOS-like rats and obese rats represented the obesity phenotype and exhibited an increase of WAT and a decrease in scapular BAT, and EA reversed this situation. Rats at the age of 21 days were implanted with DHT to induce a PCOS-like phenotype, and the other group was fed a high-fat diet to induce an obese phenotype. After 8 weeks of DHT treatment, rats in the PCOS + EA and obesity + EA groups received EA treatments for 4 weeks. **A** Body weights of the rats in each group. The arrow indicates the beginning of the EA treatment. **B** Weights of the inguinal fat pad, mesenteric fat, and periovarian fat. **C** Weight of the anterior tibial muscle and soleus muscle. **D** Scapular BAT micro CT images. **D**’ Volume of scapular BAT. **E** Scapular BAT in each group. **E**’ Weight of scapular BAT. Results are presented as means ± SEM. *p < 0.05, **p < 0.01, ***p < 0.001 vs. Control group; #p < 0.05, ##p < 0.01, ###p < 0.001 vs. Obesity group; †p < 0.05, ††p < 0.01, †††p < 0.001 vs. PCOS group (n = 6)

To investigate the possible positive impact of EA on adipose tissue metabolism in PCOS-like rats, we first monitored the body weight change together with the distribution of adipose tissue and muscle. We found that the body weight began to increase starting from the third week after modeling in both PCOS-like and obese rats, while low-frequency EA treatment could help with the weight control (Fig. 1A). Inguinal and mesenteric fat were found to be increased significantly, and a similar therapeutic effect was seen for EA in both PCOS-like and obese rats (Fig. 1B). Periovarian fat also increased, but EA did not alter this trend in the PCOS-like rats. The muscle mass remained unchanged (Fig. 1C), indicating that EA might function through effects only on adipose tissue. Using micro-CT, BAT was classified in vivo from neighboring tissue, and its volume was quantitated, which revealed the restoration of BAT by EA in both PCOS-like and obese rats (Fig. 1D and D’). The observed changes in BAT were also consistent with the gross morphological and weight changes observed after sacrifice (Fig. 1E and E’). Taken together, both obese and PCOS-like rats showed disorders of adipose tissue, especially the reduction of BAT, and EA may improve these disorders by restoring BAT.

### Reproductive and metabolic dysfunction occurred in PCOS-like rats, while only the latter occurred in obese rats

After observing the change in adipose tissue, we further investigated the serum profiles of lipid-related metabolism and liver function. As shown in Table 2, abnormal glucose metabolism was seen in both the PCOS-like and obese rats, as reflected as an increased fasting insulin level and insulin resistance. As for the detection of dyslipidemia in both PCOS-like and obese rats, representative indices such as TG, TC, LDL, ApoE, chemerin, and FFA were increased, while HDL was decreased. Moreover, there was also a certain degree of liver dysfunction, as shown by the elevation of ALT, AST, CRP, and ALD. Interestingly, all these abnormal changes were reverted or improved with EA.

**Table 2:** Profiles of endocrine, metabolism, and liver function alterations in each group (n = 6)

	Control	Obesity	Obesity + EA	PCOS	PCOS + EA
Glucose metabolism					
Fasting insulin (uIU/ml)	15.38 ± 0.60	36.73 ± 1.75***	**25.09 ± 1.33**^**###**^	33.59 ± 2.06***	**19.65 ± 1.68†††**
Fasting glucose (mmol/l)	5.03 ± 0.09	5.14 ± 0.17	4.91 ± 0.22	5.53 ± 0.09	**4.76 ± 0.10††**
OGTT					
Glucose 30 min (mmol/l)	8.35 ± 0.70	9.09 ± 0.40	8.01 ± 0.39	7.96 ± 0.31	7.23 ± 0.21
Glucose 60 min (mmol/l)	7.78 ± 0.75	8.63 ± 0.51	8.30 ± 0.30	7.80 ± 0.22	7.65 ± 0.15
Glucose 90 min (mmol/l)	6.43 ± 0.22	8.01 ± 0.45***	7.86 ± 0.20	6.56 ± 0.18	6.63 ± 0.13
Glucose 120 min (mmol/l)	5.51 ± 0.70	7.16 ± 0.35***	7.13 ± 0.21	5.84 ± 0.15	5.49 ± 0.11
Glucose AUC	13.10 ± 0.10	15.94 ± 0.69***	15.10 ± 0.19	14.00 ± 0.31**	**13.31 ± 0.10††**
HOMA-IR	3.89 ± 0.20	9.17 ± 0.71***	**5.89 ± 0.39**^**##**^	7.89 ± 0.50***	**4.69 ± 0.37††**
Lipid profile					
TC (mmol/l)	1.90 ± 0.05	2.78 ± 0.06***	**2.27 ± 0.08**^**###**^	2.75 ± 0.07***	**2.08 ± 0.08†††**
TG (mmol/l)	0.86 ± 0.04	1.44 ± 0.03***	**1.10 ± 0.04**^**##**^	1.35 ± 0.04***	**1.01 ± 0.10††**
HDL-C (mmol/l)	0.99 ± 0.08	0.61 ± 0.05**	**0.95 ± 0.08**^**#**^	0.57 ± 0.08**	**0.95 ± 0.11†**
LDL-C (mmol/l)	0.27 ± 0.01	0.36 ± 0.04*	0.29 ± 0.04	0.41 ± 0.05*	**0.23 ± 0.04††**
Leptin (ng/ml)	1.41 ± 0.05	2.17 ± 0.12***	**1.71 ± 0.12**^**#**^	2.21 ± 0.22***	**1.61 ± 0.09†**
ApoE (ng/ml)	72.10 ± 4.74	120.45 ± 7.71***	104.05 ± 4.12	138.20 ± 6.82***	**87.09 ± 8.34†††**
FFA (nmol/ml)	300.26 ± 13.96	451.81 ± 28.97***	**362.73 ± 8.62**^**#**^	424.40 ± 30.81***	**299.69 ± 20.85†**
Chemerin (ng/ml)	3.73 ± 0.20	6.13 ± 0.21***	**4.93 ± 0.48**^**#**^	5.90 ± 0.22***	5.01 ± 0.37
Liver function					
ALT (U/L)	20.69 ± 2.01	35.22 ± 1.92***	**18.48 ± 1.84**^**###**^	32.77 ± 3.66*	**17.80 ± 3.11††**
AST (U/L)	20.04 ± 1.62	31.47 ± 1.30***	**22.89 ± 1.93**^**##**^	37.60 ± 3.06***	**20.46 ± 2.16†††**
CRP (ug/ml)	3.212 ± 0.16	5.38 ± 0.38***	4.77 ± 0.23	5.73 ± 0.15***	**4.14 ± 0.30†**
ALD (ng/L)	255.58 ± 16.30	508.22 ± 39.49***	**343.88 ± 22.15**^**##**^	475.56 ± 53.48**	**297.08 ± 22.90†**

Results are presented as means ± SEM, and multiple comparisons between different groups were performed by one-way ANOVA. The bold showed the data with significant differences compared with either control or another two non-EA group. A p-value less than 0.05 was considered statistically significant

*TC* total cholesterol, *TG* triglyceride, *HDL-C* high density lipoprotein cholesterol, *LDL-C* low density lipoprotein cholesterol, *ApoE* apolipoprotein E, *FFA* free fatty acid, *OGTT* oral glucose tolerance test, *Glucose AUC* area under curve = 0.5 × (BG0 + BG30)/2 + 0.5 × (BG30 + BG60)/2 + 0.5 × (BG60 + BG90)/2 + 0.5 × (BG90 + BG120)/2, *HOMA-IR* homeostatic model assessment for insulin resistance = fasting insulin (mU/l) × fasting glucose (mmol/l)/22.5, *ALT* alanine transaminase, *AST* aspartate transaminase, *CRP* C-reactive protein

^*^p < 0.05, **p < 0.01, ***p < 0.001 *vs*. Control; ^#^p < 0.05, ^# #^p < 0.01, ^# ##^p < 0.001 *vs.* Obesity; †p < 0.05, ††p < 0.01, †††p < 0.001 *vs.* PCOS

**Fig. 2:**
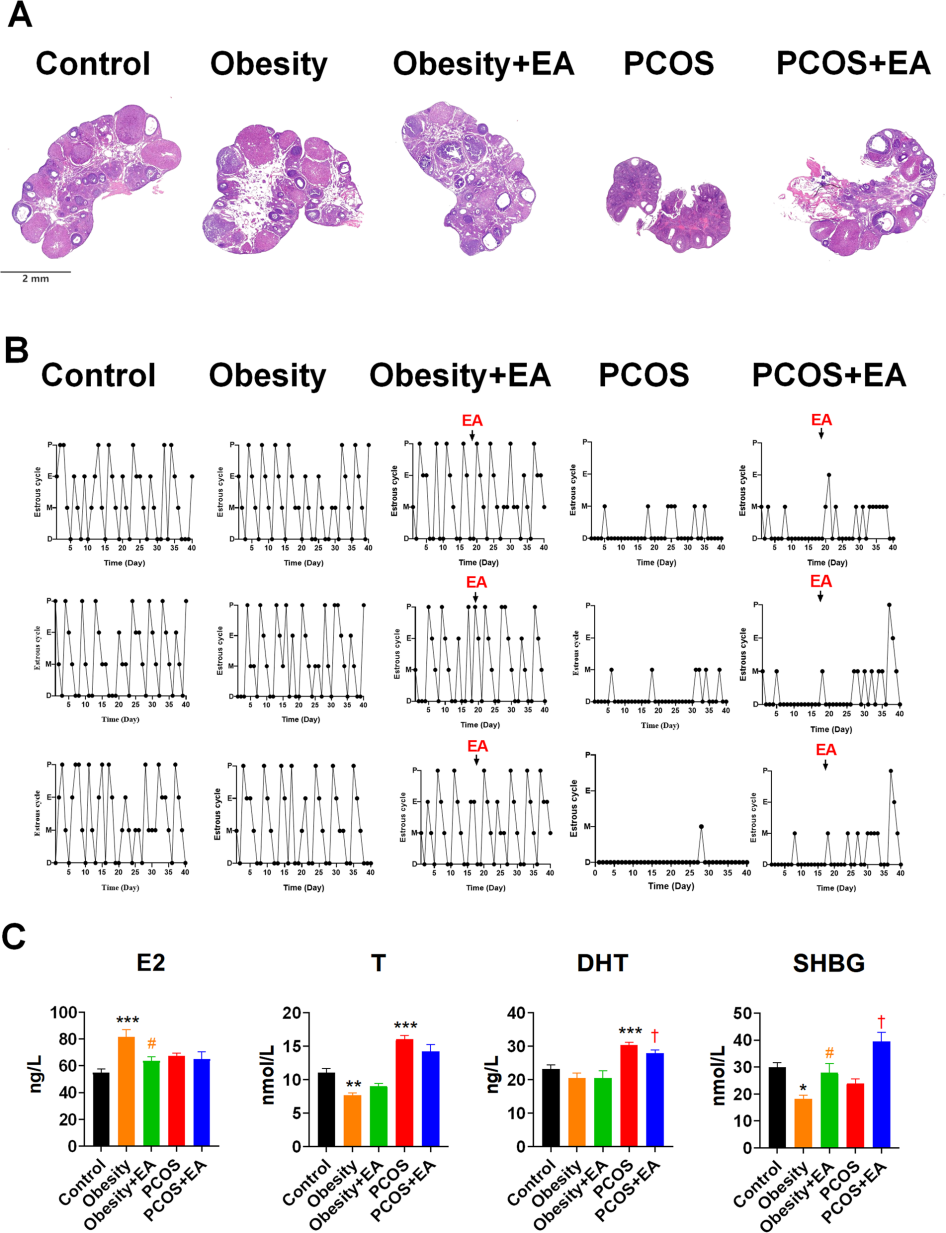
EA ameliorated PCOS acyclicity and gonadal hormone disorder. **A** H&E staining showing that the ovaries of the PCOS group were smaller than controls and had only a few corpora lutea and had more cystic follicles. **B** Estrous cycle in each group. The arrow indicates the beginning of the EA treatment. **C** Gonadal hormone profile in each group. *E2* 17β-estradiol, *T* testosterone, *DHT* dihydrotestosterone, *P4* progesterone, *SHBG* sex hormone binding globulin. Results are presented as means ± SEM. *p < 0.05, **p < 0.01, ***p < 0.001 vs. Control group; #p < 0.05, ##p < 0.01, ###p < 0.001 vs. Obesity group; †p < 0.05, ††p < 0.01, †††p < 0.001 vs. PCOS group (n = 6)

We next evaluated reproductive capacity by means of ovarian histology, cycle monitoring, and hormone detection. Reproductive disorders were only found in the PCOS-like rats, with significantly decreased numbers of corpora lutea and abnormal estrous cycles (Fig. 2A, B) as well as increased levels of T and decreased levels of SHBG (Fig. 2C). Moreover, EA could improve the reproductive parameters in PCOS-like rats, thus indicating the tandem positive effects of EA on both reproductive and metabolic dysfunction in PCOS-like rats.

### EA activated BAT sympathetic innervation and UCP1

**Fig. 3:**
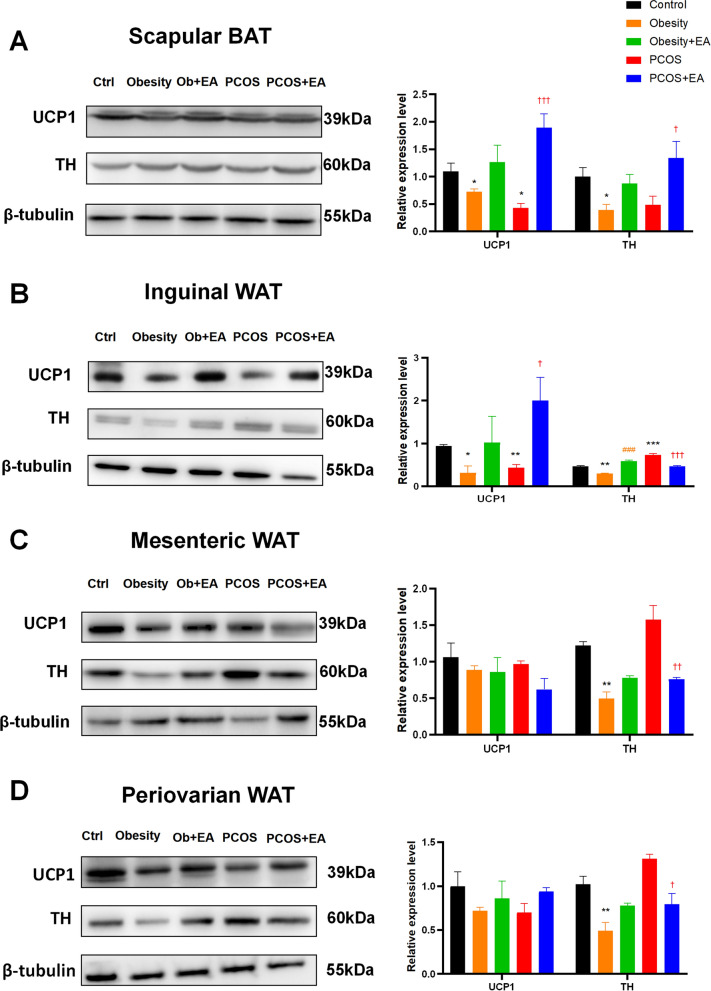
EA significantly activated scapular BAT through sympathetic stimulation in PCOS rats. (**A**–**D**) UCP1 and TH protein expression level in scapular BAT, inguinal WAT, mesenteric WAT, and periovarian WAT. Results are presented as means ± SEM. *p < 0.05, **p < 0.01, ***p < 0.001 vs. Control group; #p < 0.05, ##p < 0.01, ###p < 0.001 vs. Obesity group; †p < 0.05, ††p < 0.01, †††p < 0.001 vs. PCOS group (n = 6)

**Fig. 4:**
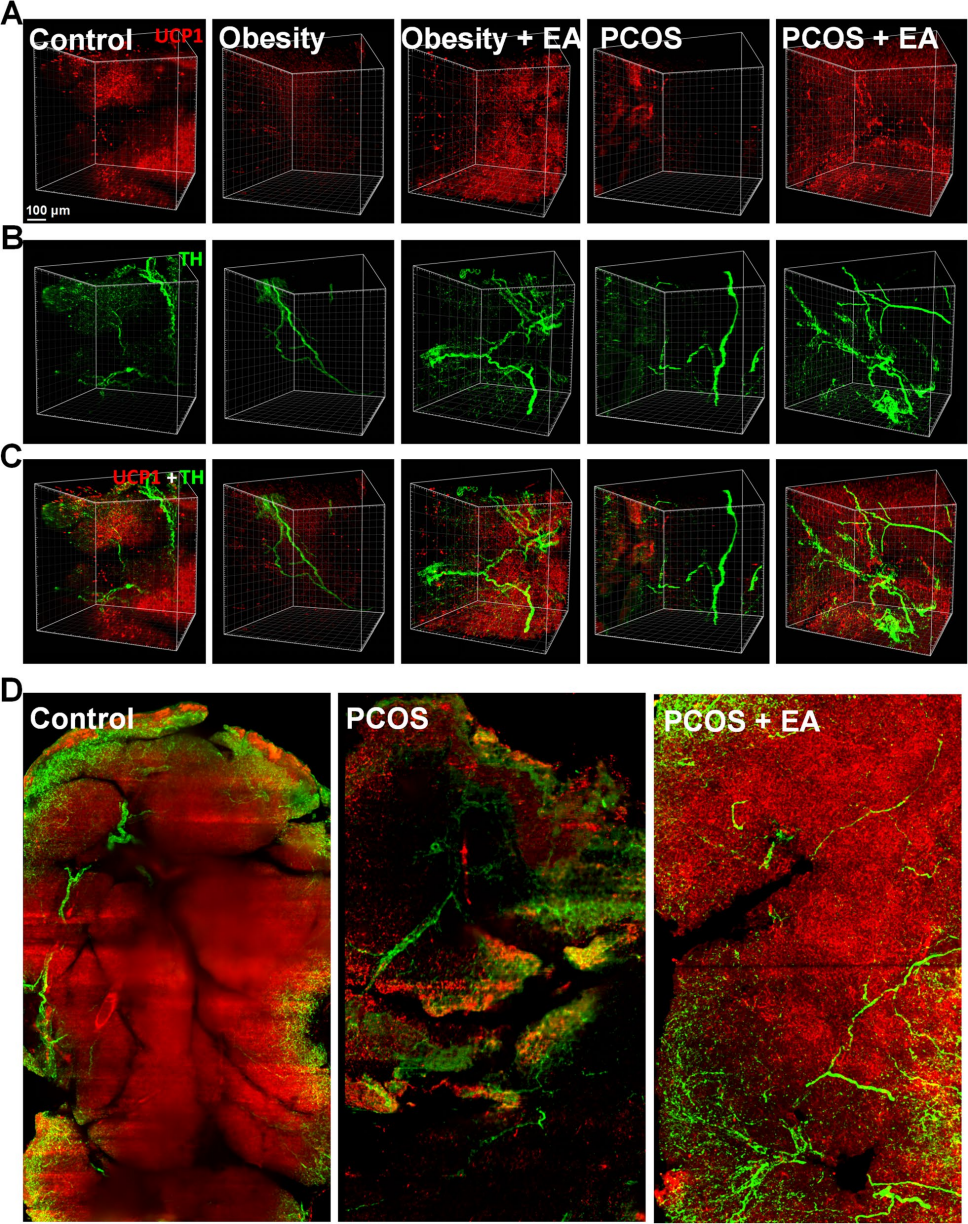
EA spatially increases the expression of UCP1 and TH in scapular BAT. **A** Expression of UCP1 (red) in each group. **B** 3D reconstruction of TH (green) in each group. **C** Representative 3D projections of BAT co-immunolabeled by TH (green) and UCP1 (red). **D** Co-localization of TH (green) and UCP1 (red) in tissue slices.

To obtain a detailed view of the connections between the sympathetic innervation and its contact with the BAT-activated marker UCP1, we took advantage of tissue clearing methods and 3D visualization technology (Additional file 2: Video S1). UCP1, an important BAT activation marker, rebounded in scapular BAT and inguinal WAT, while the sympathetic marker TH showed the same trend with UCP1 in BAT but not in WAT (Figs. 3 and 4A). Because the sympathetic nerve was in a state of hyperactivation in PCOS rats, the TH level in the PCOS group was higher than the control group (Fig. 3). And the reducing expression of TH in PCOS BAT was considered to attribute to the testosterone. High level of testosterone was proved to down-regulate the expression of UCP1 and the inhibit the activity of sympathetic nervous system in BAT [23, 24]. 3D imaging showed that the numbers of sympathetic nerves and UCP1-positive cells were decreased in the BAT of obese and PCOS-like rats, and these were restored after EA (Figs. 3A, 4A, B). When comparing the protein-expression levels of BAT activation-related UCP1 and the sympathetic marker TH, we found slight difference among EA treatment effect between PCOS-like and obese rats. This was manifested as the synchronous double elevation of UCP1 and TH after EA treatment in PCOS-like rats, but only trends for an elevation after EA treatment in obese rats (Fig. 3A), indicating the possible major involvement of sympathetic stimulation of BAT in EA’s effect on PCOS, but not in the case of obesity. Moreover, we found that UCP1 tended to accumulate at the ends of crooked sympathetic nerves (Fig. 4C). Thus, we enlarged the images and found three kinds of sympathetic nerves based on their morphological characteristics, including vascular twisted, crooked, and dispersed nerves, which could also be quantitatively classified (data not shown). Compared with the vascular twisted sympathetic nerves, the crooked and dispersed nerves were more likely to be remodeled after EA. To further define the exact relationship between UCP1 and these different kinds of sympathetic nerves, we calculated the colocalization ratio and found that EA mainly increased UCP1 in crooked or dispersed sympathetic nerves (Fig. 4D), indicating that EA might act by increasing local sympathetic innervation and thus activating UCP1 in BAT.

When turned to the change of browning-related UCP1 and sympathetic marker TH in WAT (Fig. 3B–D), and we found that although there was obvious sympathetic activation of BAT after EA treatment, no such similar sympathetic augmentation was observed in WAT. A little increase of UCP1 occurred in the inguinal WAT, indicating other mechanism underlying the effect of EA on WAT.

### EA improved chronic inflammation mainly in WAT

**Fig. 5:**
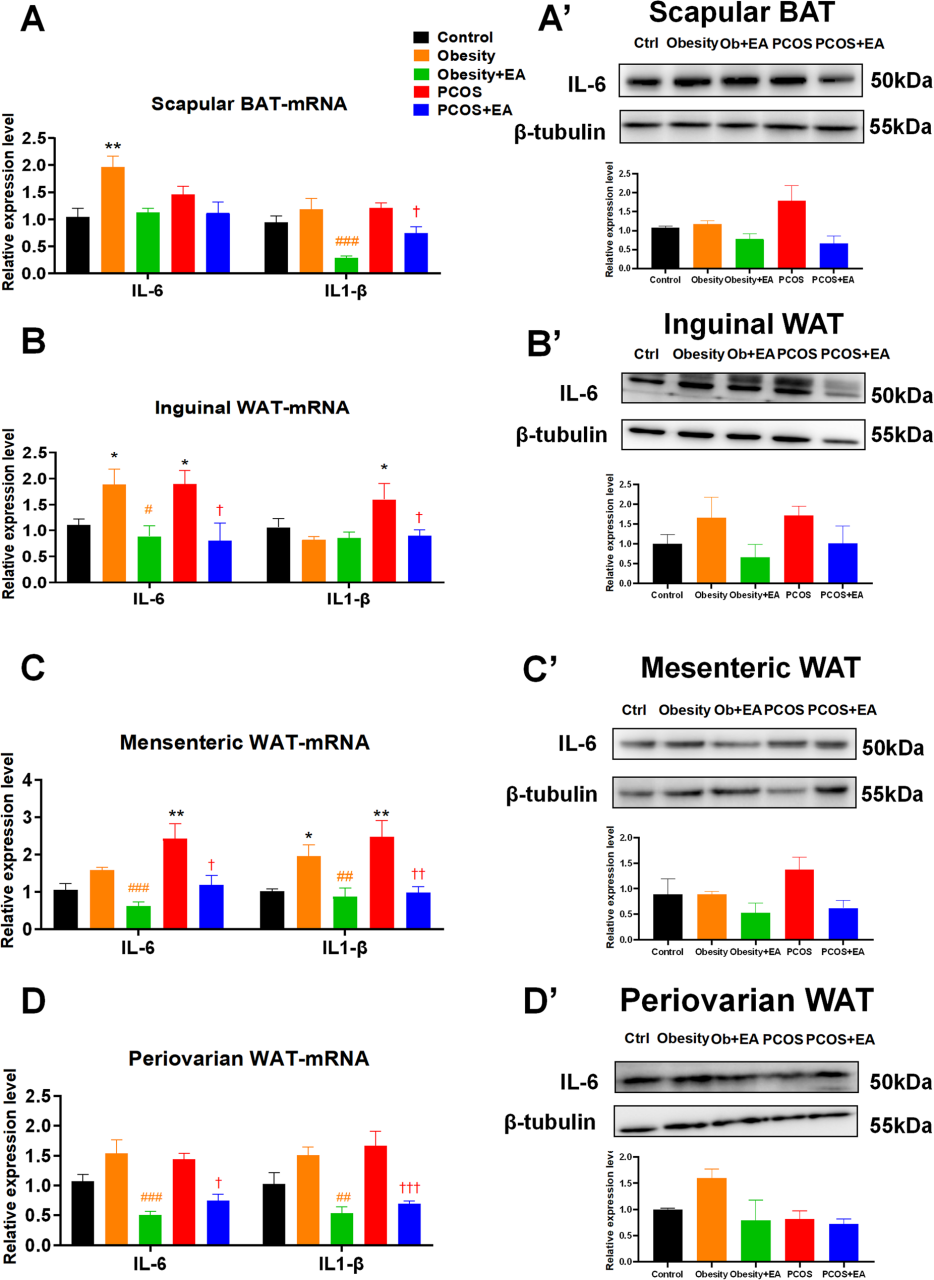
EA obviously ameliorates chronic inflammation of WAT in obese rats. **A**–**D** Transcript level of *Il-6* and *Il-1β* in scapular BAT, inguinal WAT, mesenteric WAT, and periovarian WAT. **A**’–**D**’ Protein expression level of IL-6 and IL-1β in scapular BAT, inguinal WAT, mesenteric WAT, and periovarian WAT. Results are presented as means ± SEM. *p < 0.05, **p < 0.01, ***p < 0.001 vs. Control group; #p < 0.05, ##p < 0.01, ###p < 0.001 vs. Obesity group; †p < 0.05, ††p < 0.01, †††p < 0.001 vs. PCOS group (n = 6)

In order to further explore EA’s effect between WAT and BAT, we explored the inflammatory condition in those adipose tissues represented by IL-1*β* and IL-6. At the transcription level, IL-6 was significantly increased in both BAT and WAT in obese rats, while only elevated in WAT in PCOS-like rats. Concerning the slight increase in IL-1*β* in PCOS-like inguinal WAT (Fig. 5A, B), it could be concluded that mainly IL-6 contributed to the chronic inflammatory in WAT under obesity condition. Moreover, IL-6 and IL-1*β* transcription in mesenteric and periovarian WAT was greatly decreased in both PCOS-like and obese rats after EA treatment, similar to trends in inguinal WAT (Fig. 5C, D), indicating that the improvement of chronic inflammation in WAT played a major role in EA-affected obesity. Our western blotting results for IL-6 expression in various adipose tissues also supported its important role in WAT and especially in the case of obesity (Fig. 5A’–D’).

## Discussion

In the present study, we established a diet-induced obesity model and a DHT-induced PCOS-like model and found that the obesity in both cases was characterized by massive increases in WAT and decreases in BAT. A series of indexes of serum lipid metabolism, including TG, TC, LDL, HDL, ApoE, FFA, chemerin, and leptin also confirmed the phenotype. In addition, DHT-induced PCOS-like rats exhibited anovulatory disorders and no estrous cycles. It was interesting that the weight and volume of scapular BAT in both obese and PCOS-like rats decreased significantly to half of that in the control group, and this loss could be rescued by EA treatment. We found that EA showed more extensive effects on sympathetic innervation of WAT and BAT in PCOS-like rats, while affecting inguinal WAT only in diet-induced obese rats. EA also activated UCP1 in BAT and inguinal WAT, and 3D imaging analysis showed the coexistence of sympathetic innervation and UCP1. In addition, the fat inflammatory reaction related to obesity was mainly seen in WAT. EA had a certain anti-inflammatory effect at the level of mRNA, but the inflammatory reaction in BAT was not obvious.

Obesity occurs when energy intake exceeds energy expenditure, and obesity increases the risk of developing diseases like dyslipidemia, insulin resistance, and hypertension. Adipose tissues play an important role in regulating systemic energy levels, especially BAT, which converts chemical energy into heat. Compared with WAT, the amount of BAT is lower and decreases with age [25]. However, long-term cold exposure can stimulate the proliferation and differentiation of mature BAT precursors, increase UCP1 expression, and increase the volume of BAT and heat production [26]. It has been reported that BAT transplantation into recipient mice increases glucose tolerance, increases insulin sensitivity, decreases body weight, and decreases fat mass, and the metabolic effects of transplantation are further improved when increasing the quantity of BAT that is transplanted [27]. Thus, based on the significant changes in BAT seen in our studies, EA might be another stimulus to active BAT, but the mechanism behind this remains unclear.

Obesity and PCOS are characterized by sympathetic nerve overactivity, which is reflected as metabolic alterations [28]. In particular, a positive relationship between sympathetic nerve activity and T levels has been demonstrated in PCOS [29, 30]. In the diet-induced obesity model, the distribution of WAT sympathetic innervation is negatively correlated with obesity, and cold stimulation can improve the metabolic disorder of obesity by activating WAT sympathetic innervation and promoting its beiging [31]. Similar sympathetic activation might also exist in BAT, mainly through the release of norepinephrine β-Adrenoceptor 1-3 to activate UCP1 and thus exert its thermogenic function. However, parasympathetic nerves are only distributed in a very small region of BAT, and their function remains unclear [32].

The innervation in tissues or whole organs is difficult to study by traditional techniques because of the small size, abundance, and irregular distribution of the nerve fibers, as well as their complex spatial network. Tissue clearing and 3D imaging can overcome the limitations of conventional histologic sections and can captures holistic filamentous structures such as nerves and blood vessels. In our previous study, we used CLARITY and CUBIC clearing method to show that EA might restore folliculogenesis and ovulation in PCOS by improving ovarian angiogenesis and innervation and increasing the coupling of the neovasculature [32]. Tissue clearing of adipose tissues is difficult due to the need to preserve lipid information. Therefore, using organic solvent methods, we modified immunostaining steps and optimized the 3D imaging quality of iDISCO to ensure full transparency and while at the same time protecting the inherent structures.

Using tissue-clearing methods showed that abundant sympathetic fibers in WAT are crucial for the cold-induced beiging process [18]. At the same time, other researchers have shown that the density of sympathetic nerve fibers is regulated by PRDM16 in adipocytes, suggesting that adipocytes can interact with nerves through a process monitored by factors secreted by adipocytes that transmit signals to nerve endings [31]. BAT is also innervated by the sympathetic nervous system. BAT sympathetic denervation eliminates thermogenesis by norepinephrine that is released from sympathetic nerves and binds to adrenergic receptors expressed on brown adipocytes. Recent studies showed that women with PCOS have lower BAT activity compared to controls, and BAT thermogenesis and the β-adrenoceptor-stimulated increase in UCP1 expression are negatively associated with androgen levels in PCOS [31, 33]. Similarly, transplantation of BAT into PCOS-like rats restores anovulation and the menstrual cycle, alleviates hyperandrogenism and polycystic ovary morphology, and normalizes systemic insulin sensitivity [8]. Our results are in line with these previous studies and highlight that endogenous BAT activity is closely related to the development of PCOS phenotypes and that BAT activation might provide a therapeutic option for the treatment of PCOS.

As a vital component of traditional Chinese medicine, acupuncture has a history of more than 2000 years. Acupuncture harmonizes the “Yin” and “Yang” and dredges the channels of “Qi” and “Blood” for treating diseases. Due to its safety and few side-effects, acupuncture has been popular in treating obesity and PCOS [34–36]. EA effectively ameliorates abdominal obesity and metabolic disorders in HFD-induced abdominally obese rats [37]. Clinical trials have also indicated that EA can decrease the levels of ALT, AST, and TG in persons suffering from nonalcoholic fatty liver disease [38]. Aging is characterized by an increase in adiposity and a decline in BAT activity and UCP1 expression, and EA has also been shown to significantly decrease fat mass and increase muscle mass in the elderly [39]. In this study, we chose bilateral “Sanyinjiao (SP6)”, which is located in the posterior part of the medial tibia, and bilateral “Guilai (ST29)”, which is located in the bilateral part below the umbilical for ST 29, as the acupoints in the treatment. Previous studies have shown that EA treatment at these regions could reduce the weight of inguinal [40] and visceral WAT [41]. The acupoints are located in the lower abdomen and lower limbs, but the mechanism through which EA’s effect on scapular BAT is exerted far from the selected acupoints is not fully understood. We hypothesize that EA works through the extensive nervous system. Because the role of BAT in the obesity phenotype and how low-frequency EA affects BAT remained unclear, we performed a series of further experiments. In the present study, EA showed extensive and consistent effects on WAT and BAT in obese and PCOS-like rats, which confirmed the results of previous studies. EA may activate UCP1 through the sympathetic innervation in adipose tissues, forming local high productivity areas in BAT and strengthen the bieging of WAT and accelerating energy metabolism.

Obesity is also characterized by a state of chronic inflammation in adipose tissue mediated by the secretion of a range of inflammatory cytokines. In parallel, there is increasing evidence that pro-inflammatory signals like TNF-α, IL-1β, and IL-6 represent important components of the thermogenic potential of BAT and may lead to their altered capacity for energy expenditure and glucose uptake in obesity [42]. In our present study, IL-1β and IL-6 were detected at higher concentrations in WAT, which indicated inflammatory conditions in both the PCOS-like and the obese rats. EA had anti-inflammatory effects in WAT, but the inflammatory effect in BAT was not obvious.

## Conclusion

BAT plays an important role in EA treatment of diet-induced obesity and PCOS-related obesity. For PCOS-related obesity, EA activated BAT sympathetic innervation and promoted the expression and coexistence of UCP1 to increase the high-energy regions, while EA had anti-inflammatory effects in WAT. For diet-induced obesity, the sympathetic effect of WAT was more significant.

## Supplementary Information

**Additional file 1**: **Table S1.** Sequences of primer pairs used for qRT-PCR measurement. **Table S2.** Antibodies: species, clone/catalog number, method, dilution, and source. **Table S3.** Catalogue of ELISA.

**Additional file 2**: **Video S1. **BAT of the control group stained using antibodies against UCP1 (red) or TH (green) for the identification of BAT activity and sympathetic nerves, respectively.

## Data Availability

The datasets used or analyzed during the current study are available from the corresponding author on reasonable request.

## Abbreviations

PCOS: Polycystic ovary syndrome
EA: Electroacupuncture
DHT: Dihydrotestosterone
WAT: White adipose tissue
BAT: Brown adipose tissue
3D: Three-dimensional
UCP1: Uncoupling protein 1
H&E: Hematoxylin and eosin
OGTT: Oral glucose tolerance test
ITT: Insulin tolerance test
Micro CT: Micro computerized tomography
E2: 17β-Estradiol
T: Testosterone
DHT: Dihydrotestosterone
SHBG: Sex hormone binding globulin
ALT: Alanine transaminase
AST: Aspartate transaminase
TG: Triglyceride
TC: Total cholesterol
LDL: Low-density lipoprotein
HDL: High-density lipoprotein
FFA: Free fatty acids
CRP: C-reactive protein
ALD: Aldosterone
IL-1*β*: Interleukin-1β
IL-6: Interleukin-6
