# Supplementary material for: Three-dimensional visualization of electroacupuncture-induced activation of brown adipose tissue via sympathetic innervation in PCOS rats
Source: Chin Med. 2022 Apr 18;17:48. doi: 10.1186/s13020-022-00603-w (PMC9016980; doi:10.1186/s13020-022-00603-w)
Supplement: Supplementary file 1 — Additional file 1: Table S1. Sequences of primer pairs used for qRT-PCR measurement. Table S2. Antibodies: species, clone/catalog number, method, dilution, and source. Table S3. Catalogue of ELISA. [file 13020_2022_603_MOESM1_ESM.docx]

**Table S1.** Sequences of primer pairs used for qRT-PCR measurement

| **Gene** | **Forward primer (5’-3’)** | **Reverse primer (5’-3’)** |
| --- | --- | --- |
| ***Ucp1*** | GTA CCC ACA TCA GGC AAC AG | GCA GAG ACC AAA AGG AAT CAG |
| ***Prdm16*** | TAT GCT GAG ATC CGT GAC AGA | CTG TGT TAT GTC CCT GCG ATT |
| ***IL-1β*** | GTT CTT TGA GGC TGA CAG ACC | GAT GCT GCT GTG AGA TTT GAA |
| ***IL-6*** | AAT CTG CTC TGG TCT TCT GGA | ATT GCT CTG AAT GAC TCT GGC |
| ***β-actin*** | CCT CTA TGC CAA CAC AGT | AGC CAC CAA TCC ACA CAG |

**Table S2.** Antibodies: species, clone/catalog number, method, dilution, and source

| **Antibody** | **Species** | **Dilution** | **Cat. No.** | **Company** |
| --- | --- | --- | --- | --- |
| UCP1 | Rabbit | 1:1000 | 23673-1-AP | Proteintech |
| UCP1 | Rabbit | 1:100 | ab10983 | Abcam |
| TH | Mouse | 1:1000 | 22941 | Immunostar |
| TH | Rabbit | 1:100 | ab112 | Abcam |
| IL-6 | Mouse | 1:1000 | Ab9324 | Abcam |
| Anti-Mouse IgG | Horse | 1:5000 | 7076 | Cell Signaling Technology |
| Anti-rabbit IgG | Goat | 1:5000 | L3012 | SAB |
| Alexa Fluor  647 Anti-rabbit IgG | Goat | 1:100 | 2047630 | Invitrogen |
| DyLight 550 Conjugation Lightning-Link | – | 1:500 | Ab201800 | abcam |

**Table S3. Catalogue of ELISA**

| \| **ELISA KITs** \| **Catalogue number** \| **Brand** \| \| --- \| --- \| --- \| \| Estradiol（E2) Rat ELISA Kit \| BPE30608 \| Lengton，Co., Ltd.,Shanghai \| \| Testoterone (T) Rat ELISA Kit \| BPE30610 \| Lengton，Co., Ltd.,Shanghai \| \| Dihydrotestosterone (DHT) Rat ELISA Kit \| BPE30472 \| Lengton，Co., Ltd.,Shanghai \| \| Sex hormone binding globulin (SHBG) Rat ELISA Kit \| BPE30368 \| Lengton，Co., Ltd.,Shanghai \| \| Low-density lipoprotein(LDL)（Colorimetry） \| BPC176 \| Lengton，Co., Ltd.,Shanghai \| \| Triglyceride（Colorimetry） \| BPC209 \| Lengton，Co., Ltd.,Shanghai \| \| High-density lipoprotein(HDL)（Colorimetry） \| BPC177 \| Lengton，Co., Ltd.,Shanghai \| \| Total cholesterol（Colorimetry） \| BPC207 \| Lengton，Co., Ltd.,Shanghai \| \| Aspartate transaminase (AST/GOT) Kit (Colorimetry) \| C010-1-1 \| Jiancheng Bioengineering Institute, Nanjing \| \| Alanine transaminase (ALT/GPT) Kit (Colorimetry) \| C009-3-1 \| Jiancheng Bioengineering Institute, Nanjing \| \| Apolipoprotein E(apo-E100) Rat ELISA Kit \| BPE30407 \| Lengton，Co., Ltd.,Shanghai \| \| Adiponectin(ADP) Rat ELISA Kit \| BPE30584 \| Lengton，Co., Ltd.,Shanghai \| \| C-reactive protein (CRP) Rat ELISA Kit \| BPE30459 \| Lengton，Co., Ltd.,Shanghai \| \| Aldosterone (ALD) Rat ELISA Kit \| BPE30586 \| Lengton，Co., Ltd.,Shanghai \| \| Leptin (LEP) RatELISA kit \| BPE30492 \| Lengton，Co., Ltd.,Shanghai \| \| Insulin (INS) Rat ELISA Kit \| BPE30620 \| Lengton，Co., Ltd.,Shanghai \| |
| --- | --- | --- | --- | --- | --- | --- | --- | --- | --- | --- | --- | --- | --- | --- | --- | --- | --- | --- | --- | --- | --- | --- | --- | --- | --- | --- | --- | --- | --- | --- | --- | --- | --- | --- | --- | --- | --- | --- | --- | --- | --- | --- | --- | --- | --- | --- | --- | --- | --- | --- | --- |
